# Thiotepa-induced toxic erythema of chemotherapy: A case report and review of the histological features

**DOI:** 10.1016/j.jdcr.2024.09.015

**Published:** 2024-10-05

**Authors:** Afua Ofori-Darko, Alison M. Treichel, Kord Honda, Timmie R. Sharma

**Affiliations:** a Case Western Reserve University School of Medicine, Cleveland, Ohio; b Department of Dermatology, University Hospitals Cleveland Medical Center, Cleveland, Ohio

**Keywords:** chemotherapy, dermatopathology, graft vs host disease, pediatric dermatology, Stevens-Johnson syndrome, thiotepa, toxic epidermal necrolysis, toxic erythema of chemotherapy

## Introduction

Thiotepa toxic erythema is a rare cutaneous adverse event associated with thiotepa, a chemotherapeutic agent used in various malignancies.^1^ Characterized by hyperpigmented patches, edematous plaques, erythema, and blistering, it predominantly affects occluded skin areas due to thiotepa excretion through sweat glands. We present a case of thiotepa toxic erythema, wherein clinical/histological findings mimicked other serious conditions such as Stevens-Johnson syndrome/toxic epidermal necrolysis (SJS/TEN) and graft-versus-host disease (GVHD), while providing a literature review of histological findings for this rare cutaneous adverse event.

## Case

An 11-year-old female underwent an autologous stem cell transplant following recurrent medulloblastoma. She was admitted for a second autologous stem cell transplant. As a part of her conditioning regimen, she received high dose thiotepa with carboplatin. She also received acetaminophen, diphenhydramine, mannitol, hydrocortisone, ondansetron, omeprazole, and lorazepam.

**Fig 1:**
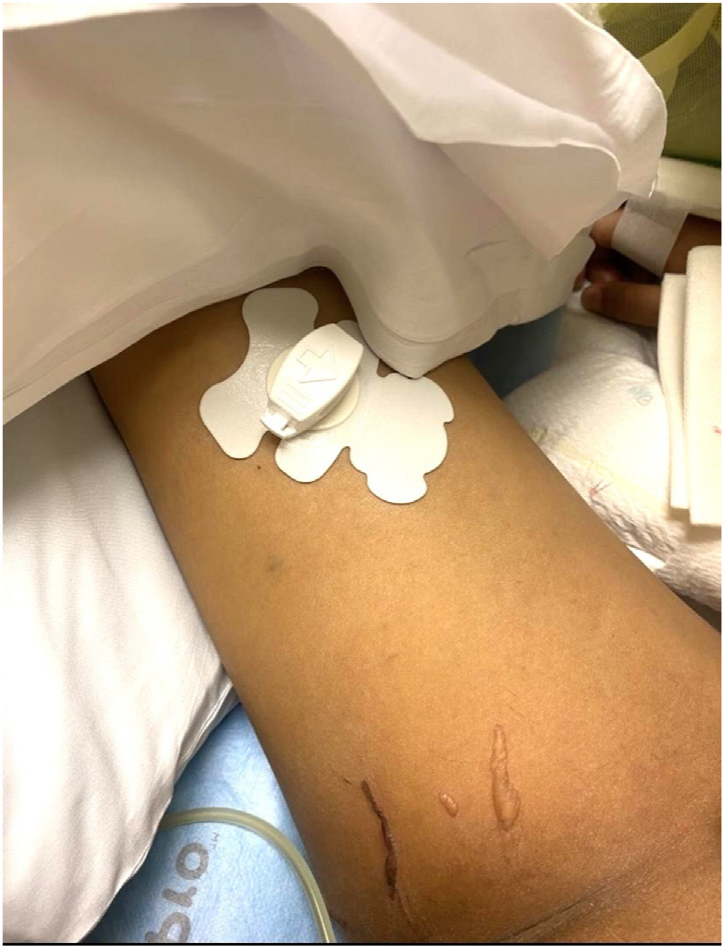
Thiotepa toxic erythema: Presence of scattered 3 cm × 3 mm linear tense bullae on the right medial thigh, underneath where a urinary catheter lay. These bullae in areas of friction/occlusion are suggestive of thiotepa toxic erythema.

**Fig 2:**
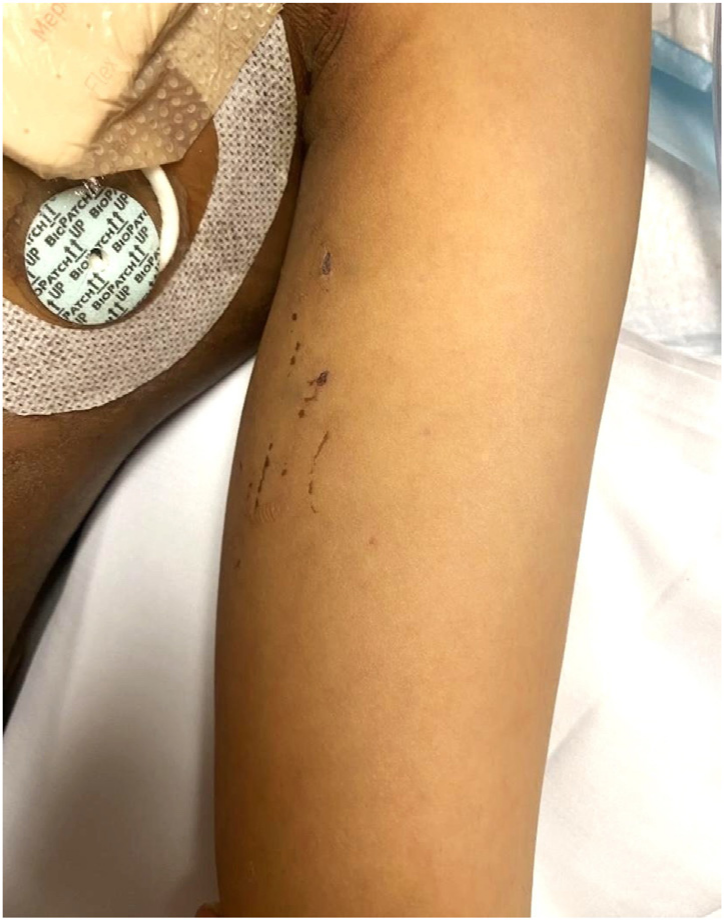
Thiotepa toxic erythema: Presence of 1-3 cm linear erosions with hemorrhagic crust on the left medial arm. The location of erosions in intertriginous sites and areas of friction is consistent with thiotepa toxic erythema.

Twelve days post-transplant, she developed a blistering rash, and dermatology was consulted due to concern for SJS/TEN. Examination revealed scattered nonerythematous tense bullae and erosions on the upper extremities, intertriginous hyperpigmented patches, and linear tense bullae on the medial thigh under the urinary catheter (Figs 1 and 2). There was no fever or mucosal involvement. A punch biopsy of a tense bulla on the right medial thigh displayed superficial epidermal necrosis with rare dyskeratosis. The clinical presentation of tense blisters below sites of friction, hyperpigmented patches in intertriginous areas, and the histologic findings were characteristic of thiotepa toxic erythema.^1^ Treatment with Vaseline application without discontinuation of her current medications was recommended and her cutaneous findings gradually resolved.

## Discussion

Thiotepa toxic erythema is a drug-specific version of toxic erythema of chemotherapy that presents similarly to malignant intertrigo, where the cutaneous findings favor intertriginous sites, but characteristically can also involve nonintertriginous sites of friction or occlusion. Thiotepa, a lipophilic alkylating agent, is used in high doses as a preparative regimen for stem cell transplantation in pediatric populations, with limited adult use at standard doses.^2^, ^3^, ^4^ It undergoes rapid hepatic metabolism but may be excreted through skin in patients receiving high-dose therapy. This can result in thiotepa toxic erythema in nearly 80% of patients, characterized by hyperpigmentation, pruritus, blistering, desquamation, and peeling, often more severe in groin, axillae, skin folds, and occluded skin.^2^^,^^4^ Thiotepa toxic erythema is often clinically diagnosed, with biopsy supporting clinical suspicion. Literature review revealed only 2 reported biopsies, both characterized by interface dermatitis, dyskeratosis, and epidermal necrosis (Table I). Thiotepa toxic erythema is self-limited, not typically requiring therapy discontinuation. Treatment focuses on symptomatic control and wound care. Showering/bathing with water and changing bed sheets/occlusive dressings at least twice daily through 48 hours after thiotepa administration is recommended to avoid thiotepa toxic erythema.^1^^,^^4^

**Table I:** Summary of histological findings of thiotepa toxic erythema reported in the literature

Case	Biopsy location	Keratinocyte necrosis	Dyskeratosis	Interface dermatitis	Other features	Reference
1	Mid back and thigh	x	x	x	Epidermal dysmaturation and foci of keratinocyte necrosis	1
2	Not documented	x	x	x	Sparse superficial perivascular mononuclear infiltrate^5^	^5^
3	Right medial thigh	x	x	x	Orthokeratosis, basal layer vacuolization	Reported case

Distinguishing toxic erythema of chemotherapy from other epidermal injury disorders is essential for accurate diagnosis and appropriate treatment. In the context of an acute blistering eruption, SJS/TEN was also considered in the differential. However, this was thought to be less likely as SJS/TEN often involves the head and neck, with severe mucositis, fever, and dusky, targetoid lesions, or widespread sloughing, none of which were present in our patient.^6^ It is also not typically limited to intertriginous folds or areas of occlusion. SJS/TEN is characterized by full-thickness epidermal necrosis in the acute phase. While superficial epidermal necrosis could be seen in resolving SJS/TEN, whereby re-epithelialization would lead to normal-appearing epidermis underneath a layer of epidermal necrosis, this did not align with the acute nature of the eruption in our patient.^1^

GVHD was also considered on the differential of an acute blistering eruption post-transplant. However, in acute GVHD, there is often involvement of acral sites, folliculocentric accentuation, and systemic mucosal involvement, which were spared in our patient.^1^^,^^7^^,^^8^ In addition, the degree of epidermal necrosis seen in our patient would only be seen in grade 3 to 4 GVHD, which would not be expected to resolve promptly without treatment. GVHD is a frequent complication in transplant patients, but its occurrence is rare in autologous stem cell transplant recipients, making this further less likely in our patient.^9^

Given the predilection for intertriginous areas, symmetrical drug-related intertriginous and flexural exanthema (SDRIFE) was considered on the differential. Similarly to thiotepa toxic erythema, SDRIFE typically spares the palms, soles and mucosa. SDRIFE is typically confined to the gluteal, perianal, inguinal, and intertriginous areas while thiotepa toxic erythema can affect any occluded or intertriginous area, as seen in the presented patient.^10^ Additionally, SDRIFE has a shorter latency period of hours to days after systemic drug exposure, compared to the longer latency period of thiotepa toxic erythema (average of 6.5 days).^2^ Histopathologic features of SDRIFE typically include a superficial perivascular infiltrate of mononuclear cells, sometimes accompanied by neutrophils and eosinophils.^10^ The presence of superficial epidermal necrosis would be unusual for SDRIFE.

In conclusion, we present an example of thiotepa toxic erythema whereby awareness and recognition of the condition can help avoid misdiagnosis of patients whose differential diagnosis could include SJS, GVHD, or SDRIFE, and avoid unnecessary drug cessation.

## Conflicts of interest

None disclosed.
